# Extracellular Vesicle-Derived microRNAs of Human Wharton’s Jelly Mesenchymal Stromal Cells May Activate Endogenous VEGF-A to Promote Angiogenesis

**DOI:** 10.3390/ijms22042045

**Published:** 2021-02-19

**Authors:** Cinzia Maria Chinnici, Gioacchin Iannolo, Ettore Cittadini, Anna Paola Carreca, David Nascari, Francesca Timoneri, Mariangela Di Bella, Nicola Cuscino, Giandomenico Amico, Claudia Carcione, Pier Giulio Conaldi

**Affiliations:** 1 Fondazione Ri.MED, Regenerative Medicine and Biomedical Technologies Unit, IRCCS-ISMETT (Istituto Mediterraneo per i Trapianti e Terapie ad Alta Specializzazione), 90127 Palermo, Italy; apcarreca@fondazionerimed.com (A.P.C.); ftimoneri@fondazionerimed.com (F.T.); mdibella@fondazionerimed.com (M.D.B.); gamico@fondazionerimed.com (G.A.); ccarcione@fondazionerimed.com (C.C.); 2 Department of Research, IRCCS-ISMETT, 90127 Palermo, Italy; giannolo@ismett.edu (G.I.); ncuscino@ismett.edu (N.C.); pgconaldi@ismett.edu (P.G.C.); 3 Casa di Cura Candela, 90141 Palermo, Italy; info@ettorecittadini.it; 4 McGowan Institute for Regenerative Medicine, University of Pittsburgh, Pittsburgh, PA 15219, USA; DGN3@pitt.edu

**Keywords:** Wharton’s jelly, mesenchymal stromal cells, extracellular vesicles, microRNAs, *VEGF-A* and *THBS1* target genes, in vitro angiogenesis

## Abstract

Despite low levels of vascular endothelial growth factor (VEGF)-A, the secretome of human Wharton’s jelly (WJ) mesenchymal stromal cells (MSCs) effectively promoted proangiogenic responses in vitro, which were impaired upon the depletion of small (~140 nm) extracellular vesicles (EVs). The isolated EVs shared the low VEGF-A profile of the secretome and expressed five microRNAs, which were upregulated compared to fetal dermal MSC-derived EVs. These upregulated microRNAs exclusively targeted the *VEGF-A* gene within 54 Gene Ontology (GO) biological processes, 18 of which are associated with angiogenesis. Moreover, 15 microRNAs of WJ-MSC-derived EVs were highly expressed (Ct value ≤ 26) and exclusively targeted the thrombospondin 1 (*THBS1*) gene within 75 GO biological processes, 30 of which are associated with the regulation of tissue repair. The relationship between predicted microRNA target genes and WJ-MSC-derived EVs was shown by treating human umbilical-vein endothelial cells (HUVECs) with appropriate doses of EVs. The exposure of HUVECs to EVs for 72 h significantly enhanced the release of VEGF-A and THBS1 protein expression compared to untreated control cells. Finally, WJ-MSC-derived EVs stimulated in vitro tube formation along with the migration and proliferation of HUVECs. Our findings can contribute to a better understanding of the molecular mechanisms underlying the proangiogenic responses induced by human umbilical cord-derived MSCs, suggesting a key regulatory role for microRNAs delivered by EVs.

## 1. Introduction

The activation of tissue repair requires the participation of multiple biological processes such as cell recruitment, proliferation, tissue remodeling, immune reactions, apoptosis and angiogenesis [1,2]. Angiogenesis in particular plays a crucial part in the correct progression of healing, since the newly formed blood vessels provide nutrients and oxygen to the growing tissue [3]. Angiogenesis is a highly regulated dynamic process involving multiple cell types and signaling pathways. The angiogenic response has been attributed in part to several angiogenic regulators, such as growth factors and chemokines, which can also be released by mesenchymal stromal cells (MSCs) and are thought to be responsible for the clinical outcomes of MSC-based therapy [4,5,6]. A mechanism of secretion of these molecules involves their export into extracellular vesicles (EVs), important mediators of intercellular communication through the release on their cargo molecules [7,8]. MSC-derived EVs have a documented capacity to actively modulate blood vessel formation [9,10,11], as well as to promote angiogenesis in animal models of myocardial infarction [12,13,14] and wound healing [15], so that their administration is an emerging approach to treat ischemic diseases. The regulation of angiogenesis-related disorders by EVs has been attributed especially to the transfer of microRNAs (miRNAs) [16,17,18,19] that modulate the expression of angiogenic genes, such as vascular endothelial growth factor (*VEGF*), matrix metalloproteinases (*MMPs),* platelet-derived growth factor (*PDGF*), fibroblast growth factor (*FGF*) and epidermal growth factor (*EGF*) [20].

Due to the remarkable proangiogenic features of their secretome, MSCs from human umbilical cords (UC-MSCs) have been successfully used to ensure therapeutic vascularization in animal models of ischemia [21,22] and myocardial infarction [23], and even in clinical settings [24]. Nevertheless, the undetectable amount of VEGF-A in the secretome of UC-MSCs suggested the existence of a still elusive, VEGF-independent mechanism regulator of angiogenesis [25,26,27]. The release of EVs, in particular, has been proposed to give a substantial contribution to UC-MSC-induced therapeutic angiogenesis [28]. On the other hand, the role of EVs from UC-MSCs in promoting tissue regeneration has been successfully investigated in various animal models of cardiovascular diseases, spinal cord injury, skin wounds, bone repair and liver diseases [29].

Understanding the molecular mechanisms of the UC-MSC-mediated proangiogenic effect is necessary to improve the effectiveness of UC-MSC-based therapy. To gain insights into these mechanisms, we compared soluble components of Wharton’s jelly-(WJ)-MSCs with those of human fetal dermal MSCs [30]—these latter cells were previously characterized for the presence of proangiogenic soluble factors and EVs in their secretome [31,32]. Secretome from both cell types was subjected to a Luminex-based quantification to detect VEGF family members, such as VEGF-A, VEGF-D and placental growth factor (PlGF)-1 [33,34], and the chemokine stromal cell-derived factor (SDF)-1 alpha, which acts as proangiogenic stimulator in tissue repair [35,36,37]. Then, EV-depleted secretome was assessed in vitro (tube formation assay, cell migration and proliferation) to evaluate the relative contribution of EVs to its functional activity. The isolated WJ-MSC-derived EVs were characterized for size, concentration, protein marker expression and contents [38]. Analysis of EV contents included the quantification of VEGF family members and SDF-1 alpha with respect to the releasing cells. Moreover, the miRNA expression profile of EVs was conducted and miRNA target genes were predicted with a bioinformatics approach. Biological validation of the identified putative target genes was carried in vitro on human umbilical vein endothelial cells (HUVECs) following exposure to WJ-MSC-derived EVs for 72 h. Finally, EV functions were assessed in vitro in cell-based assays of angiogenesis, cell migration and proliferation. The internalization of carboxyfluorescein diacetate succinimidyl ester (CFSE)-labeled EVs by HUVECs was analyzed at confocal microscopy.

## 2. Results

### 2.1. Isolation of MSCs and Immunophenotype Analysis

**Figure 1:**
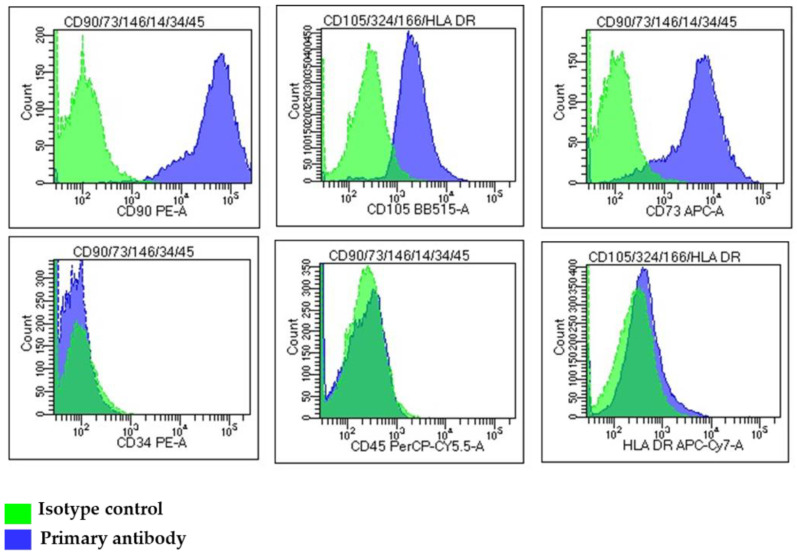
Immunophenotype analysis of cultured Wharton’s jelly-mesenchymal stromal cells (WJ-MSCs) at passage 3. Flow cytometry histograms of a representative WJ-MSC sample showing the expression of MSC markers CD90, CD105 and CD73, hematopoietic markers CD34 and CD45, and the immunoreactivity marker HLA-DR. Green color: isotype control; purple color: primary antibody.

The first sign of cell outgrowth from umbilical cord biopsies occurred at day 10–15. After reaching 80% confluence, MSCs were transferred to 75 cm^2^ plastic tissue flasks and subcultured every 4 days (split ratio 1:3) before undergoing growth arrest after 8–10 passages. The isolated WJ-MSCs at P3 were CD90^+^ (98.7 ± 2.3% positive cells), CD105^+^ (93.3 ± 3.2% positive cells) and CD73^+^ (99 ± 2.3% positive cells), while they lacked the expression of CD34, CD45 and HLA-DR surface antigens (Figure 1). UC-MSCs at passage 3 shared a similar immunophenotype (93.4 ± 3.5% CD90^+^, 83.8 ± 1.19% CD105^+^, 99.7 ± 1.19% CD73^+^, CD34^−^, CD45^−^ and HLA-DR^−^).

### 2.2. Quantitative Evaluation of the Secretome of Fetal Dermal MSCs, WJ- and UC-MSCs

The levels of VEGF-A were significantly lower in the secretome of MSCs isolated from the umbilical cord (170 ± 24 pg/mL for WJ-MSCs and 180 ± 18 for UC-MSCs, respectively) compared to the secretome of fetal dermal MSCs (5600 ± 150 pg/mL). Chemokine SDF-1 alpha was detected at a similar amount in the secretome of all the assessed cell types, but was slighter higher in UC-MSC secretome (Table 1). VEGF-D and PlGF-1 were undetected in the secretome of all the assessed cell types. The contents of VEGF-A and SDF-1 alpha detected in EVs of MSCs from umbilical cord (both UC and WJ) were similar to those of the releasing cells (Table 1).

### 2.3. In Vitro Angiogenesis Induced by Secretome and EVs of WJ-MSCs

**Figure 2:**
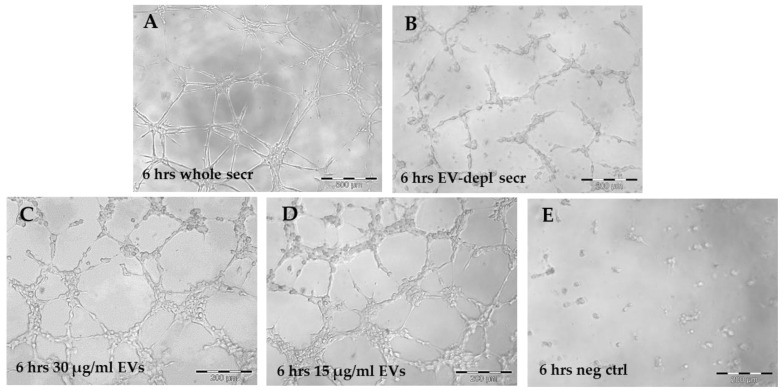
In vitro angiogenesis (tube formation assay) induced by WJ-MSC secretome and EVs. (**A**) Human umbilical-vein endothelial cells (HUVECs) in secretome 6 h after plating onto matrigel. (**B**) HUVECs in EV-depleted secretome 6 h after plating onto matrigel. (**C**) HUVECs treated with 30 μg/mL EVs 6 h after plating onto matrigel. (**D**) HUVECs treated with 15 μg/mL EVs 6 h after plating onto matrigel. (**E**) Negative control, HUVECs in serum-free culture medium 6 h after plating onto matrigel. Scale bars: 500 µm (**A**); 200 µm (**B**–**E**). Abbreviations: h, hours; secr, secretome; depl, depleted; EV, extracellular vesicle; neg ctrl, negative control.

**Figure 3:**
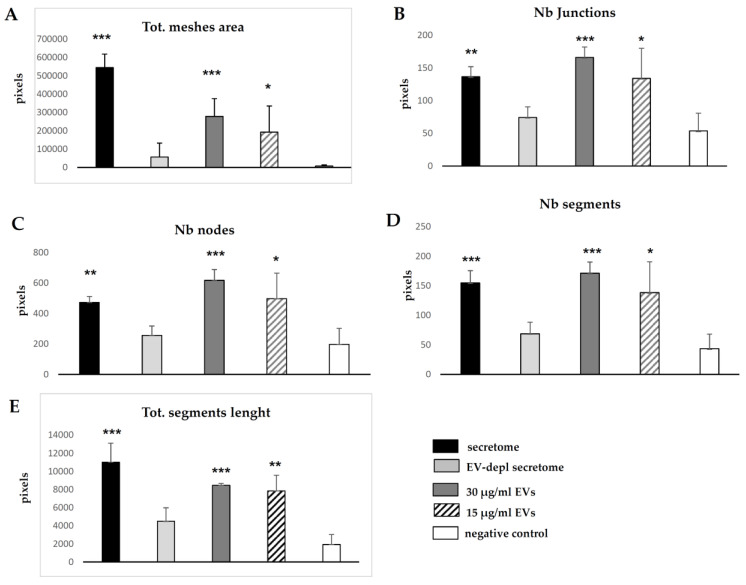
Angiogenic parameters quantified with the Angiogenesis Analyzer of ImageJ on images indicating: (**A**) total mesh area, (**B**) number of junctions, (**C**) number of nodes, (**D**) number of segments, (**E**) total segment length. Plotted values (mean ± SD) represent samples (*n* = 3 for each condition, except *n* = 4 for control). * *p* ≤ 0.05, ** *p* ≤ 0.001, *** *p* ≤ 0.0001 significant vs. negative control. Differences not denoted with an asterisk are not significant. Abbreviations: Tot, total; Nb, number; EV, extracellular vesicle; depl, depleted.

HUVECs cultured in the presence of the secretome of WJ-MSCs achieved the “complex mesh-like structures develop” pattern (maximum score 5) 6 h after plating onto matrigel (Figure 2A), while they achieved the “sprouting of new capillary tubes visible” pattern (score 3) when cultured in the presence of EV-depleted secretome (Figure 2B). Adding 30 or 15 μg/mL EVs to HUVECs stimulated the “close polygons begin to form” pattern (score 4) (Figure 2C,D). Negative control HUVECs in serum-free culture medium retained the “individual cells well separated” pattern (score 0) (Figure 2E). Quantification of angiogenic parameters by Angiogenesis Analyzer of ImageJ confirmed the extent of tube formation determined using microscope (Figure 3).

### 2.4. Characterization of WJ-MSC EVs by Nanoparticle Tracking Analysis (NTA) and Western Blot

**Figure 4:**
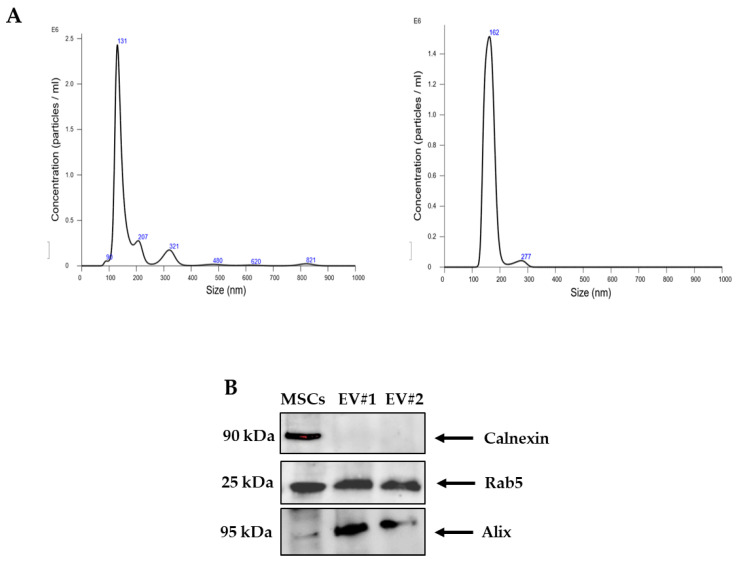
Characterization of WJ-MSC-derived EVs by NTA and Western blot analysis. (**A**) Representative NTA graphs of two EV samples (#1 and #2) obtained after differential ultracentrifugation of secretome, showing size diameter (nm) and concentration (particles/mL) of particle populations. (**B**) Representative Western blot analysis showing the expression of Calnexin, Rab4 and Alix proteins in total protein extracts from one MSC sample and two EV samples. Calnexin was used to discriminate EVs from the EV-releasing cells, WJ-MSCs. Abbreviations: NTA, nanoparticle tracking analysis; EV#1, extracellular vesicle sample #1; EV#2, extracellular vesicle sample #2.

The nanoparticle tracking analysis (NTA) of pellet particles derived from the differential ultracentrifugation of the WJ-MSC secretome revealed a homogeneous population with an average size of ~140 nm, which were defined as “small” EVs in accordance with the literature [38] (Figure 4A). The concentration of these EVs was in a range of 10^7^–10^11^ particles/mL. Biochemical composition revealed that EVs express Rab5 and Alix proteins, while they are negative for Calnexin—the latter is found in the releasing cells (Figure 4B; Figure S1). 

### 2.5. Differential Expression of EV-Derived miRNAs and Their Association with GO Biological Processes Related to Angiogenesis and Tissue Repair

Nine miRNAs (let-7f, 27b-3p, 125a-5p, 126-3p, 137, 146a, 335, 369-5p and 539) were found significantly upregulated in EVs from WJ-MSCs compared to EVs from fetal dermal MSCs (Table S1), with five miRNAs (146a-5p, 27b-3p, 137, 125a-5p and 126-3p) targeting the *VEGF-A* gene within 54 Gene Ontology (GO) terms (Table S2). Eighteen out of 54 GO terms were associated with angiogenesis (Table 2; Table S2). In addition, 24 miRNAs were found highly expressed (Ct values ≤ 26) in WJ-MSC EVs (Table 3; Table S1), 15 of which (let-7b-5p, let-7e-5p, 21-5p, 99a-5p, 100-5p, 125b-5p, 127-3p, 145-5p, 193b-3p, 199a-3p, 214-3p, 221-3p, 222-3p, 320a and 484) targeted the thrombospondin 1 (*THBS1*) gene within 75 GO terms (Table S3). Among the 75 predicted GO terms, 30 were associated with regulation tissue repair (Table 4; Table S3).

### 2.6. Effect of the WJ-MSC Secretome and EVs on the Migration and Proliferation of HUVECs

**Figure 5:**
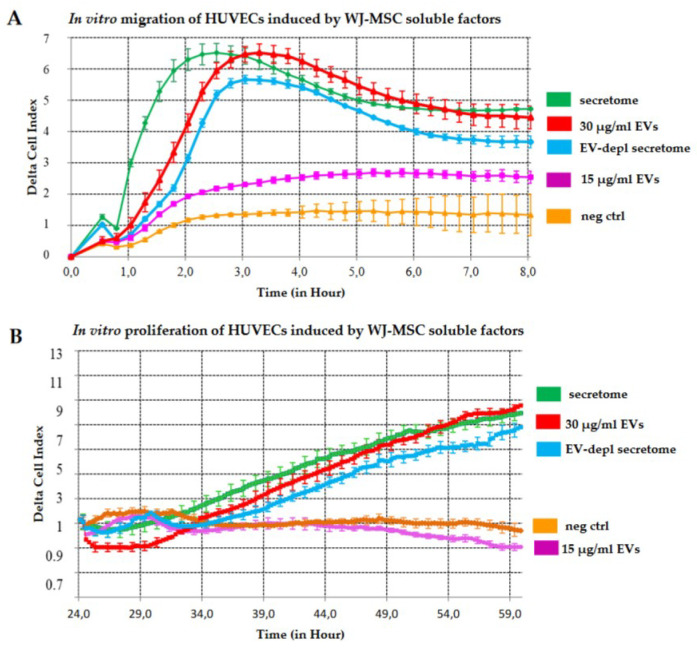
In vitro migration and proliferation of HUVECs induced by WJ-MSC secretome and EVs. (**A**) Real-time migration of HUVECs monitored with xCELLigence for 8 h and expressed as delta cell index. (**B**) Real-time proliferation of HUVECs monitored with xCELLigence for 60 h and expressed as delta cell index. Green curve: whole secretome; blue curve: EV-depleted secretome; red curve: 30 μg/mL EVs; pink curve: 15 μg/mL EVs; orange curve: serum-free culture medium (negative control). Abbreviations: EVs, extracellular vesicles; depl, depleted; neg ctrl, negative control.

Whole secretome, 30 μg/mL EVs or EV-depleted secretome were equally effective in inducing the migration of HUVECs (Figure 5A). The migratory response was significantly reduced when using lower EV concentrations (15 μg/mL) (Figure 5A). The proliferation of HUVECs was induced in a similar manner by whole secretome, 30 μg/mL EVs or EV-depleted secretome, while it was abrogated when using lower EV concentrations (15 μg/mL) (Figure 5B).

### 2.7. Validation of miRNA Putative Target Genes, VEGF-A and THBS1 by Luminex and Western Blot

**Figure 6:**
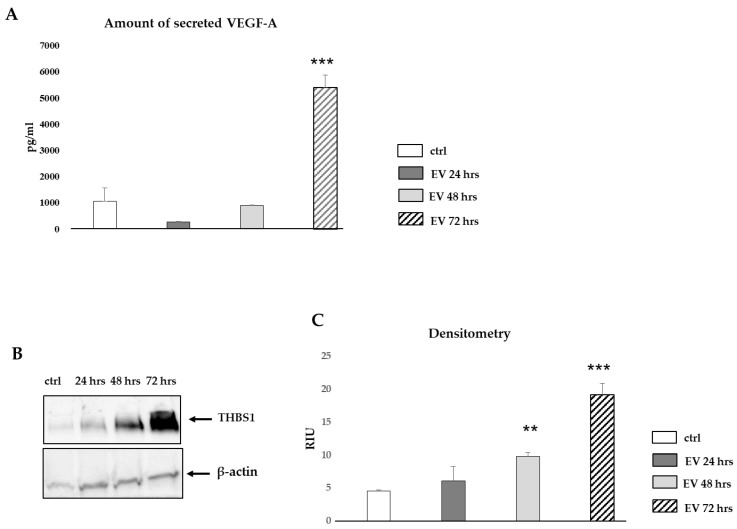
Biological validation of miRNA target prediction analysis. (**A**) Luminex quantification of VEGF-A in secretome of HUVECs untreated or treated with WJ-MSC-derived EVs for 24, 48 and 72 h. Plotted values represent mean ± SD (*n* = 3 samples); *** *p* < 0.00005 significant 72 h vs. negative control. Differences not denoted with an asterisk are not significant. (**B**) Representative Western blot showing THBS1 protein expression in HUVECs untreated or treated with WJ-MSC-derived EVs for 24, 48 and 72 h. β-actin was used as internal loading control. (**C**) Densitometric analysis of Western blot. Plotted values represent mean ± SD (*n* = 3 samples, except for *n* = 2 samples for the 48-h time point); ** *p* < 0.0005 significant 48 h vs. negative control; *** *p* < 0.00005 significant 72 h vs. negative control. Differences not denoted with an asterisk are not significant. Abbreviations: ctrl, control; h, hours; EV, extracellular vesicles; THBS1, thrombospondin 1; RIU, Relative Intensity Unit.

The levels of VEGF-A found in the HUVEC secretome following a 72 h-treatment with 30 µg/mL WJ-MSC-derived EVs were significantly higher (~5-fold increase) than those found in the secretome of the control, EV-untreated HUVECs (5397 ± 483 pg/mL vs. 1679 ± 264 pg/mL) (Figure 6A). Moreover, THBS1 protein amounts were significantly increased in HUVECs treated for 48 and 72 h (~2- and 4-fold increase, respectively, in densitometry) with 30 µg/mL WJ-MSC-derived EVs compared to the control, EV-untreated HUVECs (Figure 6B,C; Figure S2).

### 2.8. Cellular Uptake of Carboxyfluorescein Diacetate Succinimidyl Ester (CFSE)-Labeled EVs

**Figure 7:**
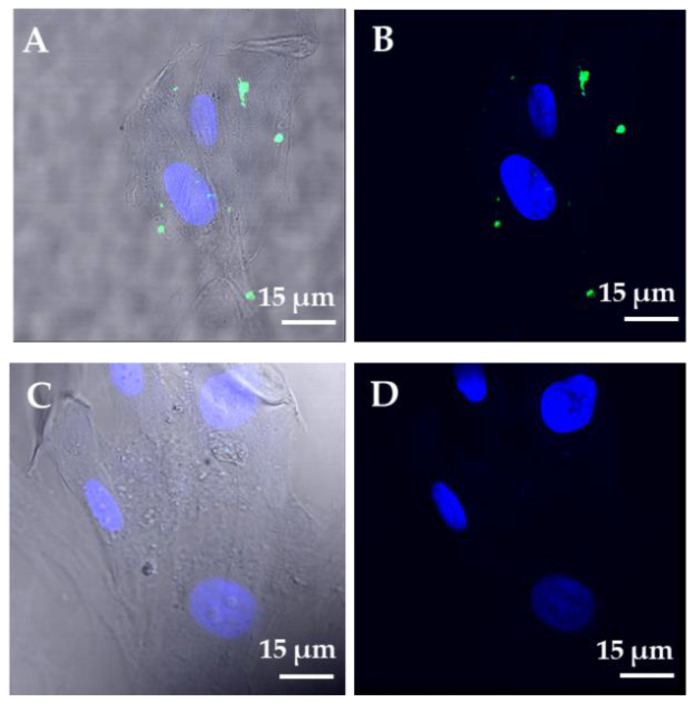
Uptake of EVs by HUVECs visualized by confocal microscopy. (**A**) Bright field image merged with green (CFSE) and blue (DAPI) showing the green fluorescent signal in the cytoplasm of HUVECs following a 4-h exposure to CFSE-labeled EVs. (**B**) Dual-channel confocal fluorescence of the same image. (**C**) Bright field image merged with green (CFSE) and blue (DAPI) showing absence of green fluorescent signal in cytoplasm of HUVECs following a 4-hour exposure to CFSE without EVs (negative control). (**D**) Dual-channel confocal fluorescence of the same image. Abbreviations: CFSE, carboxyfluorescein succinimidyl ester; DAPI, 4′,6-diamidino-2-phenylindole; EVs, extracellular vesicles. Scale bars: 15 µm.

Four hours after exposure to CFSE-labeled EVs, a punctuated green fluorescence pattern was observed in the cytoplasm of HUVECs (Figure 7A,B), while no signal was detected at earlier time points (2-h exposure; data not shown). Negative control HUVECs exposed to CFSE alone for 4 h showed an absence of green fluorescence (Figure 7C,D).

## 3. Discussion

Our laboratory is dedicated to the isolation of MSCs from different tissues, including the human placenta [51,52], the fetal liver [53] and the fetal dermis [30,32], with the aim to elucidate the cell mechanism of action and potential challenges deriving from their use in cell-based therapies. Nevertheless, the shortage of human fetuses, whose availability largely depends on therapeutic abortions, encouraged us to consider additional sources of MSCs, such as the umbilical cord discarded with the placenta after birth. MSCs were isolated from the Wharton’s jelly portion of the cord by a non-enzymatic, cell outgrowth-based technique. The immunophenotype of the isolated WJ-MSCs was similar to that of fetal dermal MSCs, herein used as the control (~ 90% CD90^+^, CD105^+^ and CD73^+^, and HLA-DR^−^, CD34^−^ and CD45^−^), indicating a quite homogeneous MSC-like phenotype [30]. In spite of lower proliferative and expansion capacities compared to control cells (outgrowth from biopsy on day 10–15 for WJ-MSCs vs. day 2 for fetal dermal MSCs; 8–10 culture passages for WJ-MSCs vs. 25–28 culture passages for fetal dermal MSCs) [30], the use of WJ-MSCs has the advantage of a greater tissue availability (an average of 26 cords/month vs. 1 fetus/month).

To verify whether our procedures ensured the collection of a secretome with proangiogenic features comparable to that of a fetal dermal MSC secretome [31,32], and also to shed a light on the molecular mechanism underlying these features, we first performed a comparative Luminex-based quantification and confirmed the low amount of VEGF-A in the secretome of WJ-MSCs reported by others [25,26,27]. In particular, we found ~150 pg/mL VEGF-A in the WJ-MSC secretome vs. ~5000 pg/mL VEGF-A in the fetal dermal MSC secretome. This latter amount is considered close to the minimum effective VEGF concentration to induce in vivo angiogenesis [36]. Some authors hypothesized that the loose connective tissue of the Wharton’s jelly may influence the weak secretion of VEGF-A from these cells [25]. Nevertheless, we tend to exclude this hypothesis since the amount of VEGF-A in the secretome of MSCs isolated from the subepithelial layer of the umbilical cord [54], and herein termed UC-MSCs, was similar to the amount found in the WJ-MSC secretome.

Assuming that a VEGF-A concentration of ~150 pg/mL may not support in vitro nor in vivo angiogenesis, the WJ-MSC secretome induced in vitro tube formation with high efficiency. This observation supports the hypothesis of a VEGF-independent pathway suggested by others [25,26,27]. Alternative pathways of angiogenesis have been more or less characterized to involve other members of the VEGF family, such as VEGF-D and PlGF, or members of the FGF family, such as PDGF-A and PDGF-C. However, none of these molecules were detected in the secretome of our WJ-MSCs. Additional proposed VEGF-independent pathways in UC-MSC secretome involve MMP-2 [26] or SDF-1 alpha [37]. For instance, it was suggested that the absence of either VEGF-A or SDF-1 can be rescued by the presence of the other one, so that the proangiogenic effect remains unchanged [37]. Nevertheless, when quantifying the amount of SDF-1 alpha in the secretome of both WJ- and UC-MSCs, we found a concentration of ~4000 pg/mL, which may not be sufficient to induce angiogenesis, requiring higher levels of SDF-1 alpha (~100 ng/mL) [55]. Consequently, we are inclined to exclude a substantial contribution of SDF-1 alpha in UC-MSC secretome-induced proangiogenic responses.

Next, we observed that the depletion of EVs significantly altered the proangiogenic activity of the WJ-MSC secretome. The isolated EVs were ~140 nm in diameter and positive for Rab5 and Alix, and negative Calnexin, thus responding to the criteria accepted by the International Society for Extracellular Vesicles to define “small” EVs [38]. Since there is no optimal isolation method, we chose differential ultracentrifugation, which is the most commonly used method for EV isolation [38]. Differential ultracentrifugation combines low- and high-speed centrifugation steps to reduce the recovery of EV subtypes, thus resulting in a preparation with intermediate specificity. Differential ultracentrifugation was always followed by NTA measurement with a NanoSight. The obtained single peaks representing the most abundant particle population suggested the absence of other EV subtypes.

With regard to EVs derived from UC-MSCs, “microvesicles” of ~ 100 nm in diameter have been indicated as key contributors to UC-MSC-induced therapeutic angiogenesis [28], but the composition of these vesicles was not analyzed. So far, there is only one published study characterizing EV contents—more precisely, the presence of angiogenic cytokines in EVs or microvesicles [56], whose delivery accounted for the UC-MSC-mediated proangiogenic effect. Our experiments of EV cargo characterization showed that the composition of the assessed soluble proteins in WJ-MSC EVs reflected those of the secretome. Interestingly, the prediction software of five miRNAs (27b-3p, 125a-5p, 146a-5p, 126-3p and 137) upregulated in WJ-MSC EVs compared to fetal dermal MSC EVs exclusively identified the *VEGF-A* target gene within GO biological processes relevant for tissue repair, such as angiogenesis, chemotaxis, differentiation, proliferation, and the inhibition of apoptosis (Table 2). Literature reports available for miR-27b-3p and miR-125a-5p [39], miR-146a [49] and miR-126 [57] support their role as angiogenesis regulators.

Target prediction analysis conducted for the highly expressed miRNAs in WJ-MSC EVs (Table 3) led to the identification of the *THBS1* gene targeted by 15 miRNAs (let-7b-5p, let-7e-5p, 21-5p, 99a-5p, 100-5p, 125b-5p, 127-3p, 145-5p, 193b-3p, 199a-3p, 214-3p, 221-3p, 222-3p, 320a and 484) within 30 GO biological processes associated with the regulation of tissue repair, including a positive regulation of angiogenesis (Table 4). THBS1 is a multifunctional extra-cellular matrix glycoprotein produced by several cell types, whose role in angiogenesis is controversial. However, evidence has recently accumulated in support of its proangiogenic functions, especially during tissue repair in different healing models [58,59,60].

Interestingly, the augmentation of both released VEGF-A and THBS1 protein expression in HUVECs treated for 72 h with WJ-MSC EVs suggests that the system “THBS1 activating VEGF activity” described by others [61] could contribute to WJ-MSC-mediated angiogenic responses. Finally, positive responses of HUVECs (in vitro tube formation, cell migration and proliferation) following treatment with WJ-MSC EVs confirmed EV functionality. The visualization of a green fluorescent signal in the cytoplasm of HUVECs following a 4-h exposure with CFSE-labeled EVs suggested that these particles are internalized by target cells.

## 4. Materials and Methods

### 4.1. Tissue Procurement, Cell Isolation and Culture

UCs were processed within 24 h after birth according to a protocol approved by ISMETT’s Institutional Research Review Board (IRRB/18/14) and Ethics Committee. A signed informed consent form was obtained from each donor. WJ-MSCs were isolated by a non-enzymatic, cell outgrowth method [30]. Briefly, after vessel removal, the UC was cut into small pieces of ~2 cm^2^ each, and plated onto 6-well sterile plastic plates (Costar Corning Inc., Costar, NY, USA) with the internal portion of WJ down. After 20 min, 1 mL of Dulbecco’s Modified Eagle Medium (DMEM) (Gibco, Thermo Fisher Scientific, Waltham, MA, USA) supplemented with 10% fetal bovine serum (FBS), l-glutamine and antibiotics (penicillin 50 IU/mL, and streptomycin 50 µg/mL) (all from Sigma-Aldrich, St. Louis. MO, USA) was carefully added on top of the pieces, which were transferred at 37°C in a humidified atmosphere with % CO_2_. Alternatively, the WJ part was removed and the pieces were plated with the external part of tight connective tissue down. Culture biopsies were checked daily for cell outgrowth from the tissue. To be distinguished from the WJ-MSCs, cells deriving from the tight connective tissue were termed umbilical cord (UC)-MSCs. Cells were harvested at 80% confluence with TrypLE (Gibco, Thermo Fisher Scientific) and transferred into 75-cm^2^ sterile tissue flasks (SARSTEDT, Numbrecht, Germany) for further expansion. Fetal skin biopsies were collected from 20- to 22-gestational-week human fetuses from therapeutic abortions, in accordance with a protocol approved by ISMETT’s IRRB (IRRB/00/15) and Ethics Committee, following a signed informed consent form from each donor. Fetal dermal MSCs were isolated, harvested and cultured as previously described [30]. HUVECs were purchased from Gibco (Thermo Fisher Scientific) and cultured in basal Medium 200 (Gibco) supplemented with low serum growth supplement (LSGS) (Gibco).

### 4.2. Immunophenotype Analysis by Flow Cytometry

The immunophenotype of WJ-MSCs and UC-MSCs was analyzed at passage 3 (P3), as previously described [30]. Briefly, cultured MSCs were harvested with TrypLE and washed in phosphate-buffered saline (PBS) without Ca^2+^/Mg^2+^ (Sigma-Aldrich). Approximately 1 × 10^6^ cells were used for each antibody staining. Acquisition of data was performed as previously described [30]. The gating strategy to distinguish cell populations was based on the physical parameters FSC vs. SSC (forward scatter vs. side scatter), excluding debris and dead cells. Then, total cell populations were visualized on the basis of single marker expression by histograms, with an overlay on the negative control (isotype) (Figure S3a–c). The used primary antibodies are listed in Table S4.

### 4.3. Harvesting of Secretome and Quantification of Soluble Factors by Luminex

The collection of secretome, including the measurement of soluble factors, was carried out as previously described [31,32]. Briefly, serum-free alpha-Minimum Essential Medium (MEM) (Gibco, Thermo Fisher Scientific) was added to 80% confluent cells, and collected 24 h later. After a centrifugation step to remove cell debris, the secretome was stored as small aliquots at −80 °C. To quantify the levels of VEGF-A, VEGF-D, PlGF-1 and SDF-1 alpha, a customized human ProcartaPlex (Invitrogen, Carlsbad, CA, USA) coupled with Luminex instrument for detection (Luminex 200; Luminex Corp. Austin, TX, USA) was used. The concentration of soluble factors was calculated with a software provided by the manufacturer and expressed as pg/mL/1.5 × 10^6^ cells/24 h, normalizing the results to the total number of the adherent cells. Luminex-based quantification of factors was also conducted on total protein extracts of EVs isolated from WJ- and UC-MSC secretomes (see below for methods).

### 4.4. In Vitro Angiogenesis Induced by Secretome and EVs of WJ-MSCs

In vitro angiogenesis was conducted as previously described [31,32]. Briefly, 10,000 HUVECs (Life Technologies, Carlsbad, CA, USA) were suspended in the secretome of WJ-MSCs and plated onto matrigel from the in vitro angiogenesis assay kit (Millipore, Billerica, MA, USA). Alternatively, HUVECs were suspended in EV-depleted secretome or in culture medium supplemented with two different concentrations of EVs (30 or 15 μg/mL, respectively). Serum-free culture medium was used as a negative control. Cells were cultured at 37 °C in a humidified atmosphere with 5% CO_2_. Tube formation was monitored under an inverted microscope, and a numerical value (score from 0 to 5) was assigned to each pattern according to the manufacturer’s specifications, and as previously described [31]. The formation of mesh-like structures was quantified by calculating the number of total mesh area, junctions, nodes, segments and total segment length with ImageJ software of the Angiogenesis Analyzer plugin (https://imagej.nih.gov/ij/).

### 4.5. Isolation of EVs and Physical Characterization by NTA

EVs were isolated by differential ultracentrifugation of the secretome of both WJ-MSCs and UC-MSCs (Optima MAX-XP, Beckman Coulter Inc., Irving, TX, USA), as previously described [32]. The size and concentration of pellet particles (*n* = 3 for each sample) were analyzed by NTA performed with a NanoSight (NS300, Malvern Instruments, Westborough, MA, USA) [62]. Briefly, five videos of each sample were recorded. Data analysis was performed with the NTA software and presented as mean ± standard deviation (SD) of the five video recordings. The resulting EV-depleted secretome was used in cell-based assays of tube formation, cell migration and proliferation.

### 4.6. Quantification of Total Protein Amounts and Western Blot Analysis

Total proteins were extracted from both cells and EVs with radioimmunoprecipitation assay (RIPA, Thermo Fisher Scientific) buffer, centrifuged at 13,000× *g* for 15 min at 4 °C, and total protein contents were quantified with the bicinchoninic acid (BCA) assay (Thermo Fisher Scientific) by using the Tecan Spark 10M microplate reader (BioExpress, VWR, Radnor, PA, USA). Thirty µg/lane of total protein extracts was separated by sodium dodecyl sulphate-polyacrylamide gel electrophoresis (SDS-PAGE). Proteins were trans-blotted onto nitrocellulose membranes (Bio-Rad Laboratories, Richmond, CA, USA). Membranes were blocked with 5% non-fat milk in Tris Buffered Saline with 0.1% Tween-20 (TBS-T) (50 mmol/L Tris pH 7.5, 0.9% NaCl, and 0.1% Tween-20) (Sigma-Aldrich), and then incubated with the following primary antibodies: mouse monoclonal antibody raised against recombinant human Rab5 (F-9, sc-373725, Santa Cruz Biotechnology, Santa Cruz, CA, USA; 1:500 dilution), mouse monoclonal antibody against Alix (3A9, 2171, Cell Signaling Technology, Danvers, MA, USA; 1:1000 dilution), and rabbit monoclonal antibody against Calnexin (C5C9, 2679, Cell Signaling, 1:1000 dilution). Β-actin (sc-81178, Santa Cruz Biotechnology; 1:1000 dilution) was used as loading control protein. After three washes in TBS-T, membranes were incubated with horse peroxidase (HRP)-conjugated secondary antibodies (Santa Cruz Biotechnology; 1:10,000 dilution), washed again with TBS-T and developed with enhanced chemiluminescence reagent (ECL) (Amersham, Arlington Heights, IL, USA). Densitometric analysis of Western blot was performed with the Image Lab software, version 6.0.1 (Bio-Rad Laboratories).

### 4.7. EV miRNA Expression Profile with TaqMan Low-Density Array (TLDA)

EVs obtained by differential ultracentrifugation of the secretome of WJ-MSCs and fetal dermal MSCs (*n* = 3 for each sample) were re-suspended in Qiazol (Qiagen, Hilden, Germany) and total RNA was extracted using the miRNeasy Mini Kit (Qiagen), according to the manufacturer’s instructions. The purity of isolated RNA was determined by OD_260/280_ using a Nanodrop ND-1000 (Thermo Fisher Scientific). Reverse Transcription (RT) and pre-amplification were performed using the High Capacity cDNA RT Kit (Applied Biosystems, Thermo Fisher Scientific) according to the manufacturer’s instructions. MiRNA profiling of EVs was carried out with TaqMan Array Human MicroRNA A+B cards (Life Technologies) by using the Applied Biosystems 7900 HT Real-Time PCR system. The expression level of each miRNA was determined by the equation 2^−ΔΔ*C*t^ and Student’s t-test was used to calculate the *p*-value with a threshold of ≤ 0.05. Data were considered significant at a fold change >15.

### 4.8. Gene Ontology (GO) Enrichment Analysis of EV miRNAs and Biological Validation of Putative Target Genes

MiRNA target genes were predicted using the on-line prediction software program DIANA-mirPath v.3 [63] by selecting the DIANA-TarBase v7.0, and a *p*-value of ≤0.05. The analysis was performed with the “genes intersection” option in order to identify single genes targeted by multiple miRNAs. The approach was used to screen out the differentially expressed miRNAs in EVs of WJ-MSCs vs. EVs of fetal dermal MSCs, as well as the highly expressed miRNAs (Ct value ≤ 26) in WJ-MSC-derived EVs. The bioinformatics approach included a GO enrichment analysis of biological processes.

The identified target genes were further validated by treating HUVECs with 30 µg/mL WJ-MSC EVs for 24, 48 and 72 h. The amount of VEGF-A released in the secretome of both untreated and EV-treated HUVECs was measured with Luminex. THBS1 protein expression levels in both untreated and EV-treated HUVECs were evaluated by Western blot (rabbit monoclonal antibody raised against recombinant anti-THBS1; ab267388, Abcam, Cambridge, UK; 1:1000 dilution), as described in the above subsection.

### 4.9. In Vitro Migration and Proliferation of HUVECs Induced by Secretome or EVs of WJ-MSCs

The migration of HUVECs was monitored with the xCELLigence Real-Time Cell Analyzer (RTCA) dual purpose (DP) instrument (Roche Diagnostics, Mannheim, Germany), which monitors the cellular events in real time, recording changes as electrical impedance. Briefly, whole secretome, EV-depleted secretome, different concentrations of EVs (30 or 15 μg/mL) or serum-free culture medium were added to the lower chamber of the Cellular Invasion/Migration (CIM)-plate as chemoattractant. Then, 30,000 HUVECs were loaded into the upper chamber. The assay was carried out according to the manufacturer’s instructions. The plates were assembled into the RTCA-DP instrument, and cell migration was recorded every 15 min for 8 h at 37°C in a humidified atmosphere with 5% CO_2_.

The proliferation of HUVECs was also monitored with the xCELLigence instrument. Briefly, 10,000 HUVECs were added to the electronic (E)-plate in the presence of whole secretome, EV-depleted secretome, different concentrations of EVs (30 or 15 μg/mL) or serum-free culture medium. Proliferation was recorded up to 60 h at 37 °C in a humidified atmosphere with 5% CO_2_. Migration and proliferation were expressed as delta cell index (CI), and the values were shown as mean ± SD from duplicate wells at the given time point. Analyses were performed by using the RTCA Software 1.2 of the xCELLigence system.

### 4.10. EV Labeling with Fluorescent Dye and Cellular Uptake Assay

The labeling of EVs with CFSE (Thermo Fisher Scientific) was carried out as previously described [32]. Briefly, 1:1000 diluted CFSE was added to 10 µg of EV preparation, and incubated at 37 °C for 15 min. The labeling was blocked with 1% bovine serum albumin (BSA) (Sigma-Aldrich) and the mixture was ultracentrifuged at 100,000× *g* for 70 min at 4 °C. The labeling of EVs was verified by flow cytometry, as previously described [32]. HUVECs grown at 60% confluence in 4-well glass chamber slides were incubated at 37 °C with CFSE-labeled EVs at a ratio of 1 µg EVs per 10,000 adherent cells or with CFSE without EVs (negative control). At the end of the incubation time (2 and 4 h), cells were washed twice with PBS and fixed with paraformaldehyde solution 4% in PBS for 10 min at room temperature. Nuclei were stained with DAPI (Sigma-Aldrich) and then mounted with Permafluor and a coverslip (Thermo Fisher Scientific). The cellular uptake of EVs was visualized under a Leica confocal station (Leica SP5 confocal system) mounted on a Leica DM6000 inverted microscope (Leica Microsystems Inc., Buffalo Grove, IL, USA).

### 4.11. Statistical Analysis

For immunophenotype analysis, three different samples for each cell type were used. For the quantification of soluble factors in MSC secretome with Luminex technology, six fetal dermal MSC samples, nine WJ-MSC and nine UC-MSC samples were used. For in vitro angiogenesis assay, secretome and EVs from three WJ-MSC samples were used. For NTA and miRNA studies, three WJ-MSC samples and three fetal dermal MSC samples were used. For biological validation of the bioinformatics data by Western blot and Luminex, three EV samples at each time point of treatment (24, 48 and 72 h) were used. For in vitro cell migration and proliferation, secretome or EVs from three different WJ-MSC samples were tested. Data were analyzed with R (https://cran.r-project.org) [64] and expressed as mean ± SD. Data from each group were compared by Student’s t-test, and the differences between the groups were considered significant at a *p*-value of ≤0.05.

## 5. Conclusions

The umbilical cord holds the promise of an accessible source of MSCs with the potential to reverse those clinical conditions where angiogenesis is impaired. The proangiogenic effect of UC-MSC secretome has been attributed to still elusive, VEGF-independent pathways. Overall, “small” EVs are herein described as important contributors of WJ-MSC-mediated proangiogenic responses. Moreover, the interaction between these EVs and the predicted miRNA target genes, VEGF-A and THBS1, is shown in vitro by treating HUVECs with appropriate doses of EVs. According to our hypothesis, the activation of VEGF-A and THBS1 by MSC-derived EVs may act as a molecular switch that triggers endogenous angiogenesis. Ultimately, EVs can work as a vehicle for a successful delivery of their miRNAs to treat various angiogenesis-related disorders. To date, only one study is available on the analysis of EV miRNAs of UC-MSCs and their association with tissue repair [60]. A more detailed analysis should provide additional clues to the precise mechanism of action by which these EV-miRNAs interact with target cells.

## Figures and Tables

**Table 1:** Luminex measurement of growth factor VEGF-A and chemokine SDF-1 alpha in secretome of human fetal dermal MSCs, WJ-MSCs and UC-MSCs, and in EVs from WJ- and UC-MSCs.

Soluble Factors (pg/mL/10^6^ Cells/24 h)	Fetal Dermal MSC Secretome	WJ-MSC Secretome	UC-MSC Secretome	WJ-MSC EVs	UC-MSC EVs
**VEGF-A**	5600 ± 150	170 ± 24 ***	180 ± 18 ***	100 ± 29 ***	150 ± 30 ***
**SDF-1 alpha**	3120 ± 900	4190 ± 920	5725 ± 900 *	5880 ± 120 *	5040 ± 280

Plotted values (mean ± SD) representing fetal dermal MSC samples (*n* = 6), WJ-MSC samples (*n* = 9) and UC-MSC samples (*n* = 9). * *p* ≤ 0.05 and *** *p* ≤ 0.0005 significant vs. fetal dermal MSC samples. Differences not denoted with an asterisk are not significant. Abbreviations: MSCs, mesenchymal stromal cells; WJ, Wharton’s jelly; UC, umbilical cord; EVs, extracellular vesicles; h, hours; SDF-1 alpha, stromal cell derived factor-1 alpha.

**Table 2:** Eighteen GO terms associated with angiogenesis, which were selected from 54 terms predicted for 5 microRNAs (miRNAs) (genes intersection 5) upregulated in EVs of WJ-MSCs vs. EVs of fetal dermal MSCs, and targeting the *VEGF-A* gene. Abbreviations: GO, gene ontology.

Selected GO Terms Associated with Angiogenesis (*VEGF-A* Target Gene)	log10 *p*-Value
lymph vessel morphogenesis	0.029070346
coronary vein morphogenesis	0.029070346
VEGF-activated neuropilin signaling pathway	0.029070346
positive regulation of cell proliferation by VEGF-activated platelet derived growth factor receptor signaling pathway	0.029070346
positive regulation of lymphangiogenesis	0.029070346
positive regulation of endothelial cell chemotaxis by VEGF-activated vascular endothelial growth factor receptor signaling pathway	0.029070346
coronary artery morphogenesis	0.035697463
vascular endothelial growth factor signaling pathway	0.035697463
positive regulation of vascular permeability	0.035697463
endothelial cell chemotaxis	0.035697463
positive regulation of cell migration involved in sprouting angiogenesis	0.035697463
tube formation	0.036090227
lung vasculature development	0.037567858
positive regulation of vascular endothelial growth factor receptor signaling pathway	0.042118988
induction of positive chemotaxis	0.043066423
cell migration involved in sprouting angiogenesis	0.043946017
positive regulation of blood vessel endothelial cell migration	0.044267247
cellular response to vascular endothelial growth factor stimulus	0.047669382

**Table 3:** List of 24 highly expressed miRNAs (Ct values ≤ 26) in EVs from WJ-MSCs and their documented role in angiogenesis (see also Table S1).

MiRNA Name	Role in Angiogenesis	References
hsa-let-7b-5p	it targets *VEGF* gene; validated role in angiogenesis	[39,40]
hsa-let-7e-5p	validated role in angiogenesis	[40]
hsa-miR-17-5p	angiogenesis promoter; it targets *VEGF* gene	[19,39,41]
hsa-miR-21-5p	angiogenesis promoter	[41]
hsa-miR-24-3p	validated role in angiogenesis; highly expressed by endothelial cells	[40,42]
hsa-miR-31-5p	angiogenesis promoter	[43]
hsa-miR-92a-3p	validated role in angiogenesis	[40,41]
hsa-miR-99a-5p	highly expressed by endothelial cells	[42]
hsa-miR-99b-3p	angiogenesis promoter	[44]
hsa-miR-100-5p	angiogenesis promoter	[45]
hsa-miR-106a-5p	it targets *VEGF* gene	[39]
hsa-miR-125b-5p	angiogenesis promoter; tube formation of HUVECs	[46]
hsa-miR-145-5p	angiogenesis promoter	[47]
hsa-miR-146a-3p	it targets *VEGF* gene	[48]
hsa-miR-191-5p	it may regulate the angiogenic actions of *VEGF*	[40]
hsa-miR-193b-3p	it targets *VEGF* gene	[39]
hsa-miR-199a-3p	it targets *VEGF* gene	[39]
hsa-miR-214-3p	it targets *VEGF* gene	[39]
hsa-miR-221-3p	validated role in angiogenesis	[40]
hsa-miR-222-3p	angiogenesis in wound healing; validated role in angiogenesis	[40,49]
hsa-miR-320a	it targets *VEGF* gene; validated role in angiogenesis	[39,40]
hsa-miR-484	it targets *VEGF* gene	[50]

**Table 4:** Thirty GO terms associated with tissue repair, which were selected from 74 terms predicted for 15 miRNAs (genes intersection threshold 15) highly expressed in WJ-MSC EVs and targeting the *THBS1* gene.

Selected GO Terms Associated with Angiogenesis and Tissue Repair (*THBS1* Target Gene)	log10 *p*-Value
positive regulation of transforming growth factor beta1 production	0.002834
positive regulation of transforming growth factor beta production	0.002834
positive regulation of fibroblast migration	0.002834
positive regulation of endothelial cell apoptotic process	0.002834
positive regulation of chemotaxis	0.002834
negative regulation of focal adhesion assembly	0.002834
negative regulation of cell-matrix adhesion	0.002905399
negative regulation of fibroblast growth factor receptor signaling pathway	0.002971086
negative regulation of blood vessel endothelial cell migration	0.003082249
positive regulation of blood vessel endothelial cell migration	0.003082249
negative regulation of endothelial cell migration	0.003082249
positive regulation of transforming growth factor beta receptor signaling pathway	0.003884392
negative regulation of endothelial cell proliferation	0.004108779
sprouting angiogenesis	0.00471601
positive regulation of endothelial cell migration	0.00471601
blood vessel morphogenesis	0.00471601
positive regulation of cell-substrate adhesion	0.00471601
negative regulation of extrinsic apoptotic signaling pathway	0.004811209
cellular response to growth factor stimulus	0.005970634
negative regulation of angiogenesis	0.006063442
response to mechanical stimulus	0.006624871
activation of MAPK activity	0.007836227
positive regulation of angiogenesis	0.009069898
positive regulation of cell migration	0.012958161
response to hypoxia	0.015327033
extracellular matrix organization	0.015939502
cell migration	0.015939502
cell motility	0.023452526
negative regulation of apoptotic process	0.034158772
cell adhesion	0.045940676

GO, gene ontology; *THBS1*, thrombospondin 1.

## Data Availability

The datasets used and analyzed are available from the corresponding author on reasonable request.

## Supplementary Materials

Supplementary materials can be found at https://www.mdpi.com/1422-0067/22/4/2045/s1.

## Abbreviations

WJ	Wharton’s jelly
UC	Umbilical cord
EVs	Extracellular vesicles
GO	Gene Ontology
THBS1	Thrombospondin 1
NTA	Nanoparticle Tracking Analysis
TLDA	TaqMan low-density array
RTCA	Real-time cell analyzer
CI	Cell index
RIU	Relative Intensity Unit
CFSE	carboxyfluorescein diacetate succinimidyl ester
DAPI	4′,6-diamidino-2-phenylindole
