# Supplementary material for: Extracellular Vesicle-Derived microRNAs of Human Wharton’s Jelly Mesenchymal Stromal Cells May Activate Endogenous VEGF-A to Promote Angiogenesis
Source: Int J Mol Sci. 2021 Feb 19;22(4):2045. doi: 10.3390/ijms22042045 (PMC7922033; doi:10.3390/ijms22042045)

BD FACSDiva 9.0

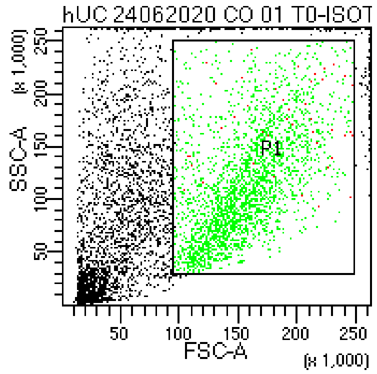

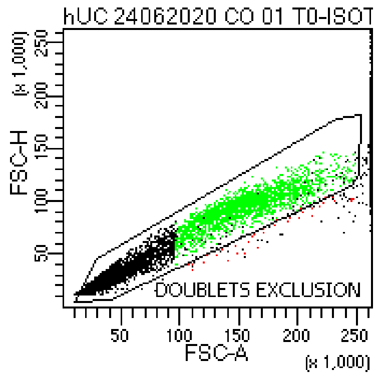

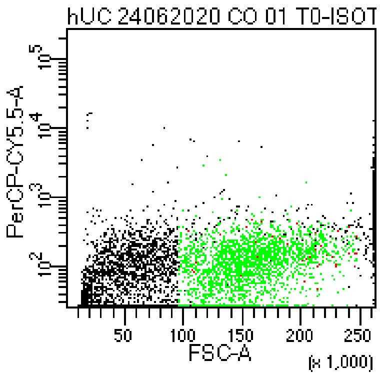

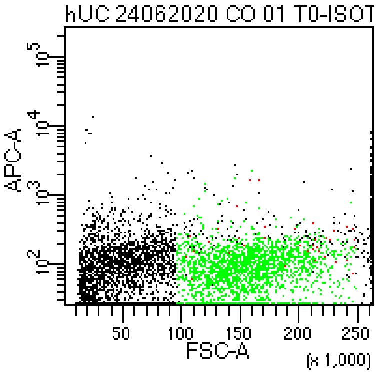

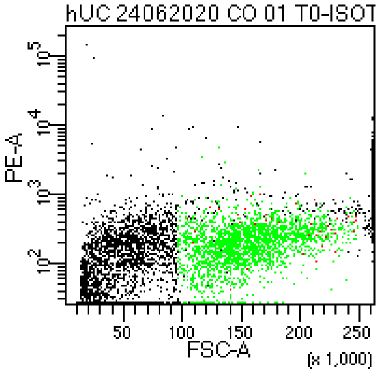

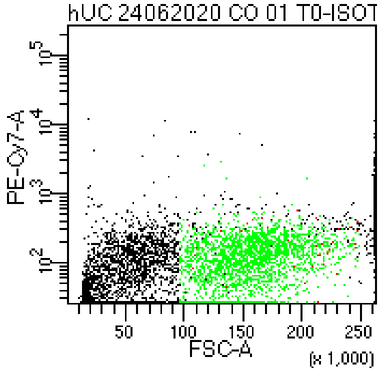

Experiment Name:	MSC
Specimen Name:	hUC 24062020 CO 01 T0
Tube Name:	ISOTYPE CTRL

Population	#Events	%Parent	FSC-A Mean	BB515-A Mean
All Events	6,003	####	117,841	446
P1	2,380	39.6	155,933	387
DOUBLETS EXCLUSION	2,328	97.8	155,270	384
Q1	11	0.5	145,600	836
Q2	1	0.0	130,181	6,338
Q3	2,311	99.3	155,337	371
Q4	5	0.2	150,311	4,008
Q1-1	0	0.0	####	####
Q2-1	4	0.2	146,397	4,631
Q3-1	2,324	99.8	155,285	376
Q4-1	0	0.0	####	####

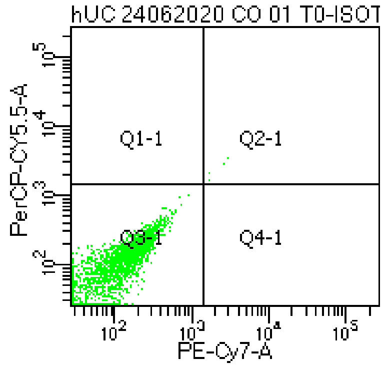

Tube: ISOTYPE CTRL

Population	#Events	%Parent	%Total
All Events	6,003	####	100.0
P1	2,380	39.6	39.6
DOUBLETS EXCLUSION	2,328	97.8	38.8
Q1	11	0.5	0.2
Q2	1	0.0	0.0
Q3	2,311	99.3	38.5
Q4	5	0.2	0.1
Q1-1	0	0.0	0.0
Q2-1	4	0.2	0.1
Q3-1	2,324	99.8	38.7
Q4-1	0	0.0	0.0

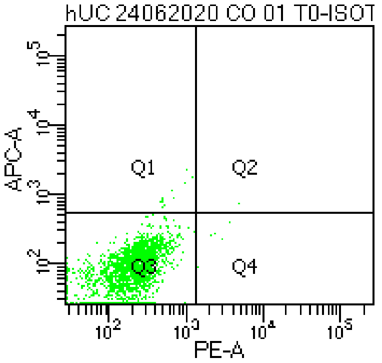

Supplement: Supplementary file 1 [file ijms-22-02045-s001.zip › Figure S3a.pdf]
