# Supplementary material for: Extracellular Vesicle-Derived microRNAs of Human Wharton’s Jelly Mesenchymal Stromal Cells May Activate Endogenous VEGF-A to Promote Angiogenesis
Source: Int J Mol Sci. 2021 Feb 19;22(4):2045. doi: 10.3390/ijms22042045 (PMC7922033; doi:10.3390/ijms22042045)

BD FACSDiva 9.0

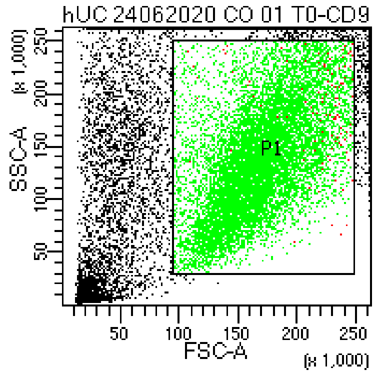

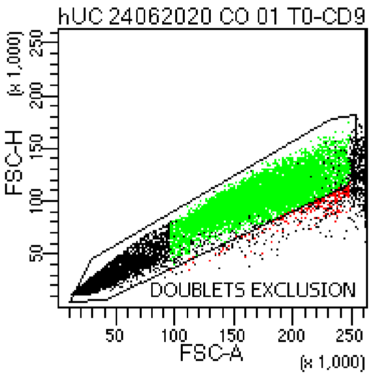

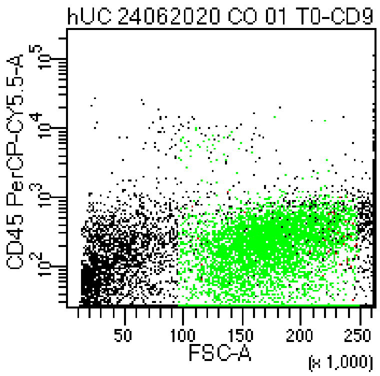

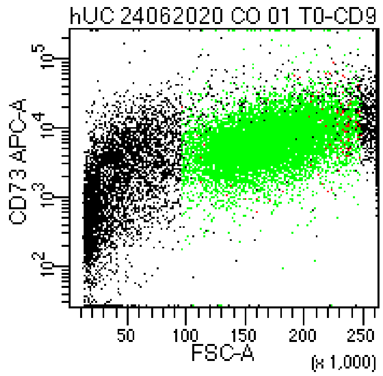

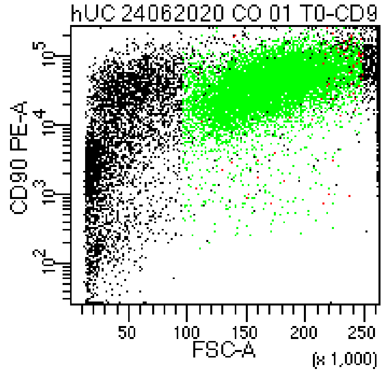

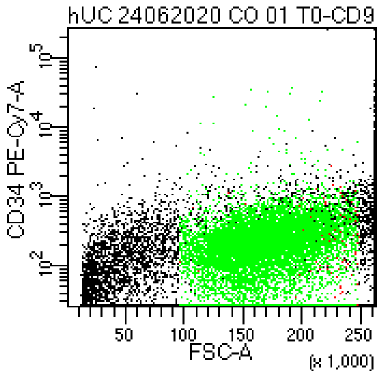

Experiment Name: MSC
Specimen Name: hUC 24062020 CO 01 T0
Tube Name: CD90/73/146/14/34/45

Population	#Events	%Parent	FSC-A Mean	CD146 ... Mean
All Events	20,000	####	194,260	3,442
P1	10,801	54.0	168,662	2,222
DOUBLETS EXCLUSION	10,577	97.9	167,726	2,188
Q1	72	0.7	150,318	1,178
Q2	10,470	99.0	167,932	2,199
Q3	12	0.1	140,149	739
Q4	23	0.2	143,194	1,239
Q1-1	41	0.4	132,991	4,243
Q2-1	6	0.1	160,341	4,264
Q3-1	10,470	99.0	167,766	2,161
Q4-1	60	0.6	185,201	5,296

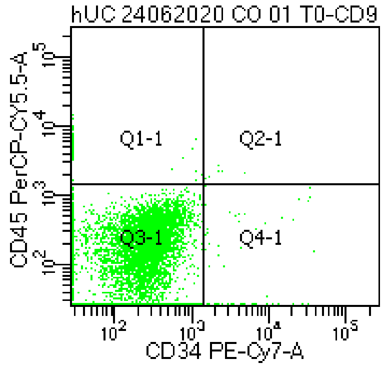

Tube: CD90/73/146/14/34/45

Population	#Events	%Parent	%Total
All Events	20,000	####	100.0
P1	10,801	54.0	54.0
DOUBLETS EXCLUSION	10,577	97.9	52.9
Q1	72	0.7	0.4
Q2	10,470	99.0	52.3
Q3	12	0.1	0.1
Q4	23	0.2	0.1
Q1-1	41	0.4	0.2
Q2-1	6	0.1	0.0
Q3-1	10,470	99.0	52.3
Q4-1	60	0.6	0.3

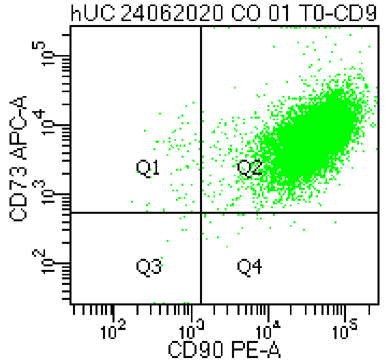

Supplement: Supplementary file 1 [file ijms-22-02045-s001.zip › Figure S3b.pdf]
