# Supplementary material for: Extracellular Vesicle-Derived microRNAs of Human Wharton’s Jelly Mesenchymal Stromal Cells May Activate Endogenous VEGF-A to Promote Angiogenesis
Source: Int J Mol Sci. 2021 Feb 19;22(4):2045. doi: 10.3390/ijms22042045 (PMC7922033; doi:10.3390/ijms22042045)

BD FACSDiva 9.0

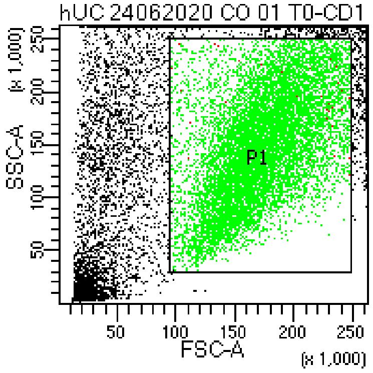

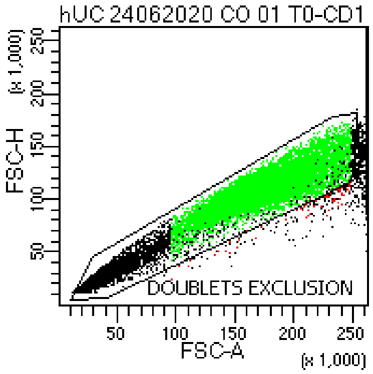

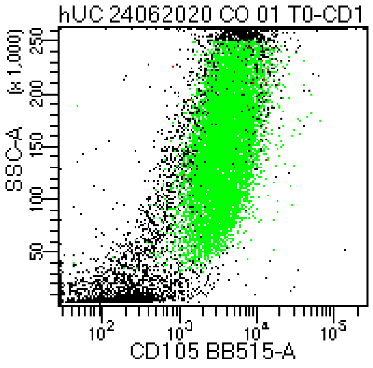

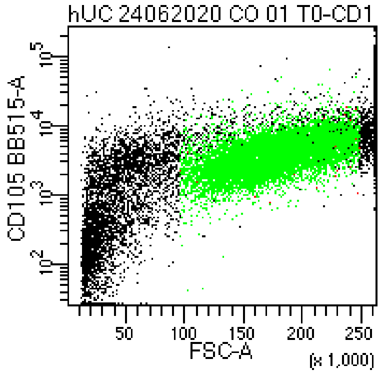

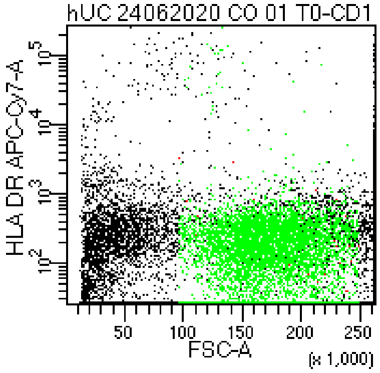

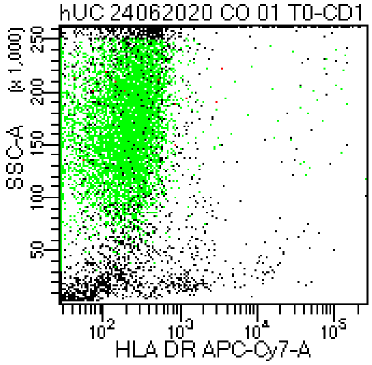

Experiment Name: MSC
Specimen Name: hUC 24062020 CO 01 T0
Tube Name: CD105/324/166/HLA DR

Population	#Events	%Parent	FSC-A Mean	CD105 ... Mean
All Events	20,000	####	210,255	6,559
P1	10,310	51.6	171,888	4,211
DOUBLETS EXCLUSION	10,239	99.3	171,643	4,195
Q1	2	0.0	103,733	692
Q2	72	0.7	164,975	6,535
Q3	83	0.8	128,038	650
Q4	10,082	98.5	172,063	4,208

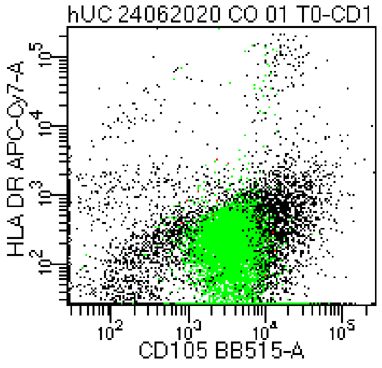

Tube: CD105/324/166/HLA DR

Population	#Events	%Parent	%Total
All Events	20,000	####	100.0
P1	10,310	51.6	51.6
DOUBLETS EXCLUSION	10,239	99.3	51.2
Q1	2	0.0	0.0
Q2	72	0.7	0.4
Q3	83	0.8	0.4
Q4	10,082	98.5	50.4

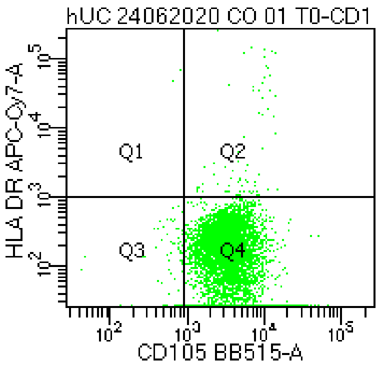

Supplement: Supplementary file 1 [file ijms-22-02045-s001.zip › Figure S3c.pdf]
