# Supplementary material for: Extracellular Vesicle-Derived microRNAs of Human Wharton’s Jelly Mesenchymal Stromal Cells May Activate Endogenous VEGF-A to Promote Angiogenesis
Source: Int J Mol Sci. 2021 Feb 19;22(4):2045. doi: 10.3390/ijms22042045 (PMC7922033; doi:10.3390/ijms22042045)
Supplement: Supplementary file 1 [file ijms-22-02045-s001.zip › Table S4.docx]

**Supplemental material S2.** Primary antibodies for flow cytometry

| **Antigen** | **Clone** | **Conjugation** | **Isotype** | **Supplier** |
| --- | --- | --- | --- | --- |
| **IgG1** | G18-145 | FITC | mouse monoclonal IgG_1_,k | BD Pharmingen  (Franklin Lake, NJ, USA) |
| **IgG2** | PC10 | PE | mouse monoclonal IgG_2_a,k | BD Pharmingen |
| **CD45** | 2D1 | APC | mouse monoclonal IgG_1_,k | BD Pharmingen |
| **CD45** | H130 | FITC | mouse monoclonal IgG1,k | BD Pharmingen |
| **CD34** | 581 | PE | mouse monoclonal IgG1,k | BD Pharmingen |
| **CD90** | 5E10 | PE | mouse monoclonal IgG_1_, k | BD Pharmingen |
| **CD105** | MEM226 | FITC | mouse monoclonal IgG_1_, k | Abcam (Cambridge, UK) |
| **CD73** | AD2 | APC | mouse monoclonal IgG_1_, k | BD Pharmingen |
| **HLA-DR** | L243 (G46-6) | PE | mouse monoclonal IgG_2a_,k | BD Biosciences |
