# Supplementary figures and images for: Extracellular Vesicle-Derived microRNAs of Human Wharton’s Jelly Mesenchymal Stromal Cells May Activate Endogenous VEGF-A to Promote Angiogenesis
Source: Int J Mol Sci. 2021 Feb 19;22(4):2045. doi: 10.3390/ijms22042045 (PMC7922033; doi:10.3390/ijms22042045)

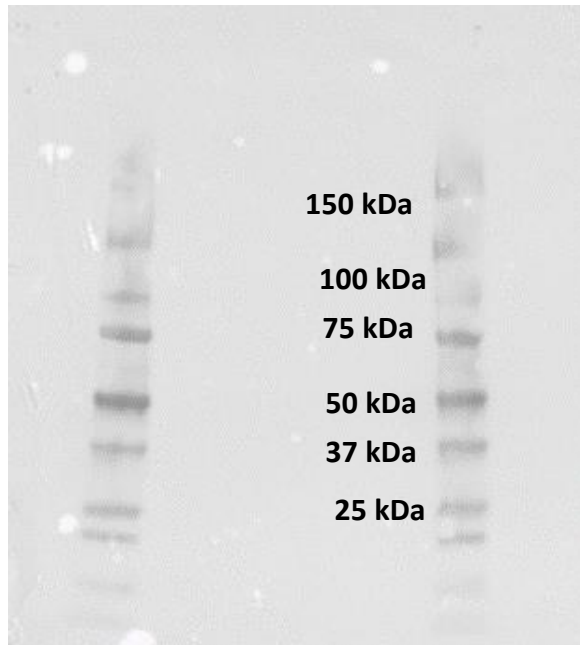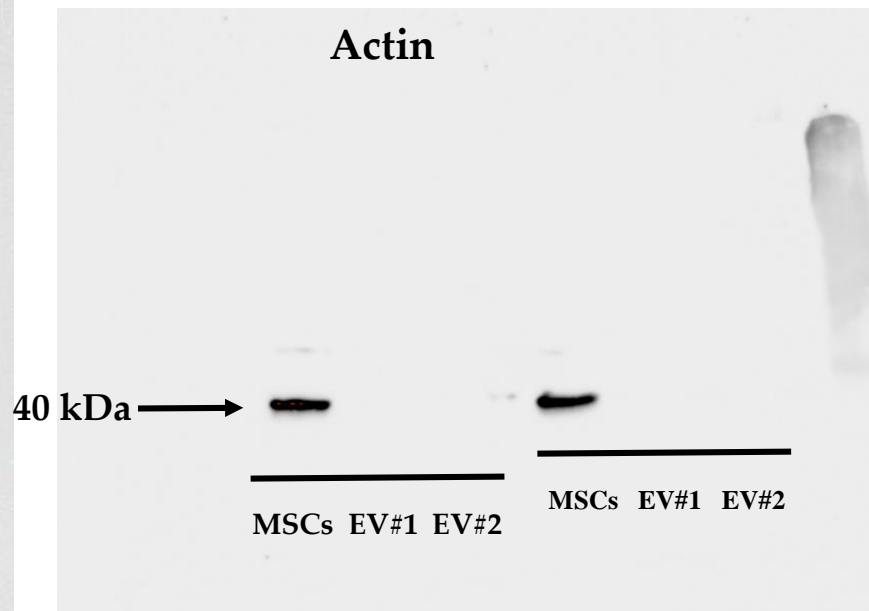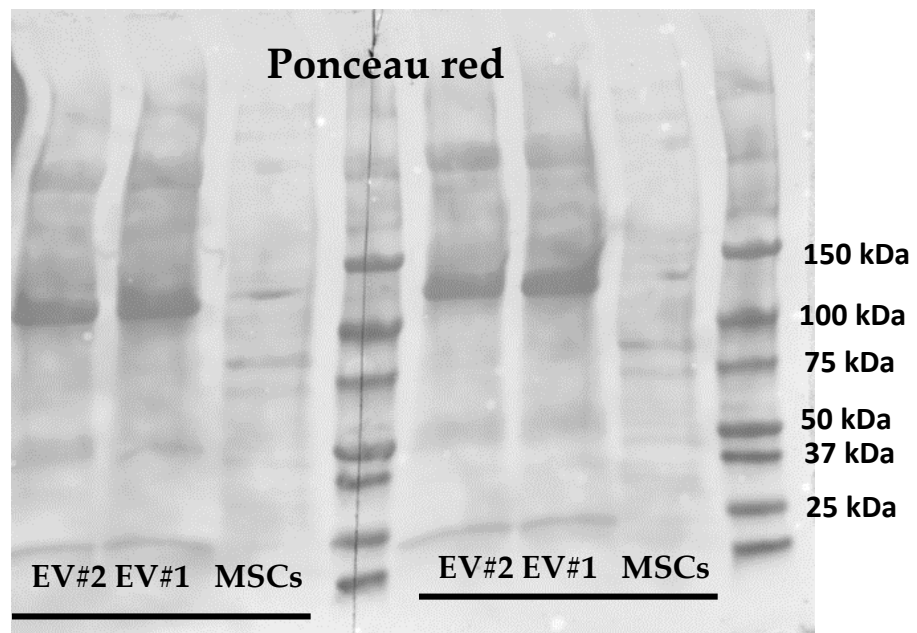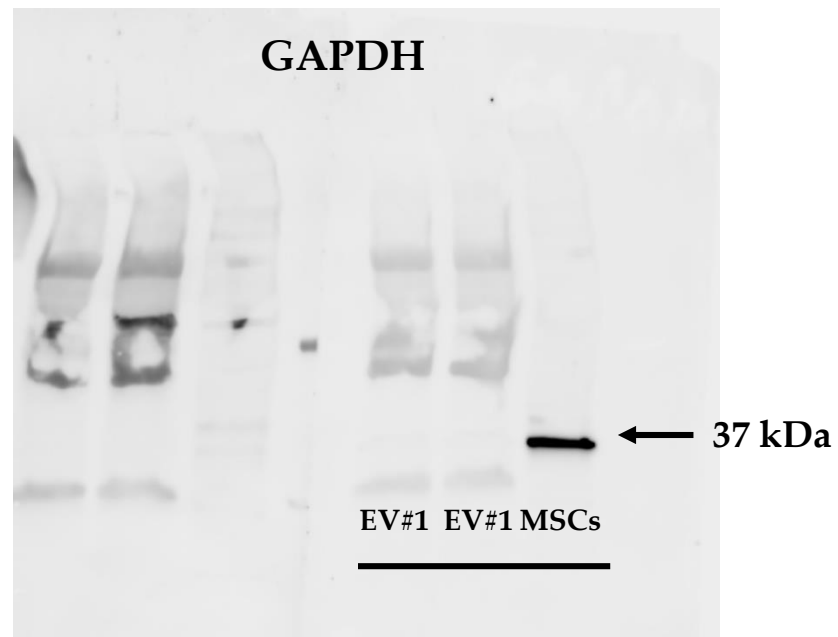

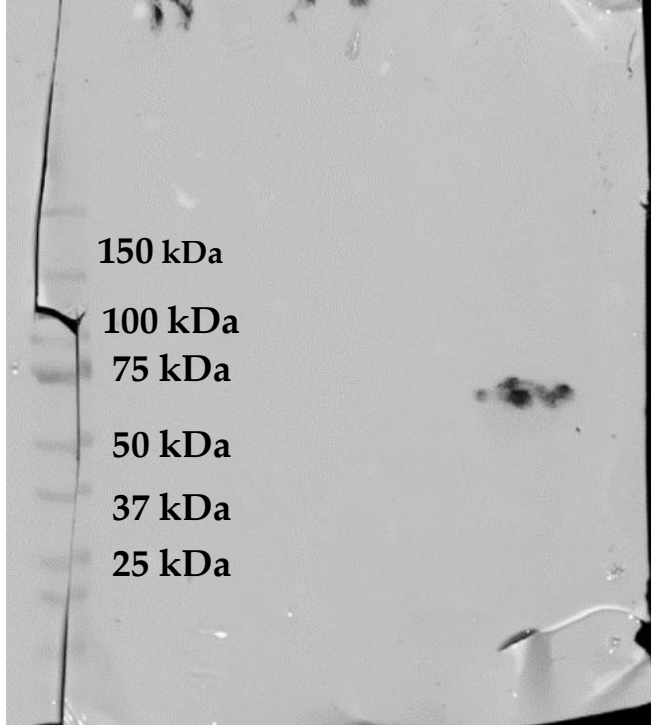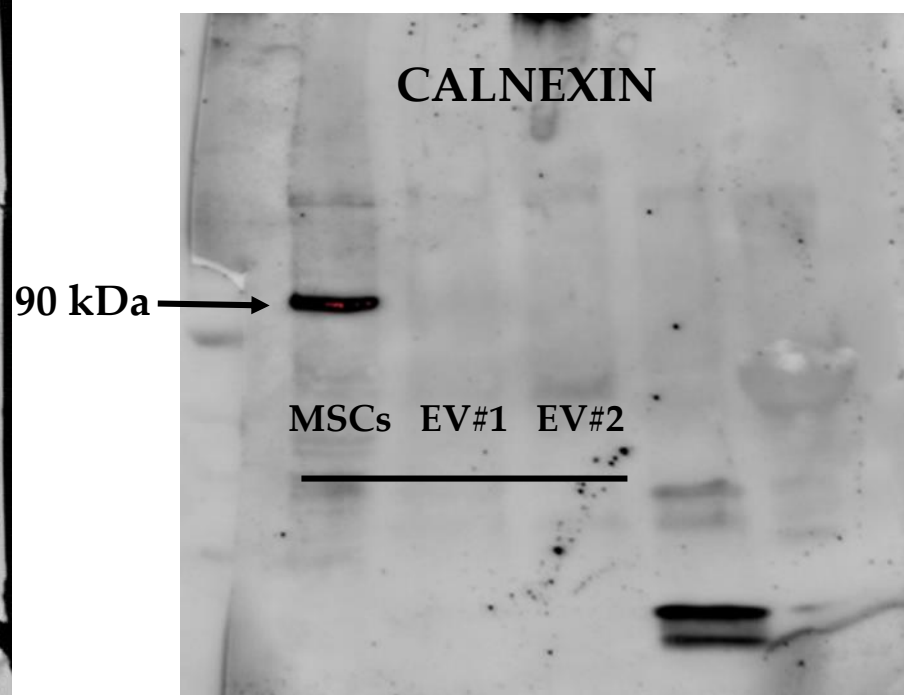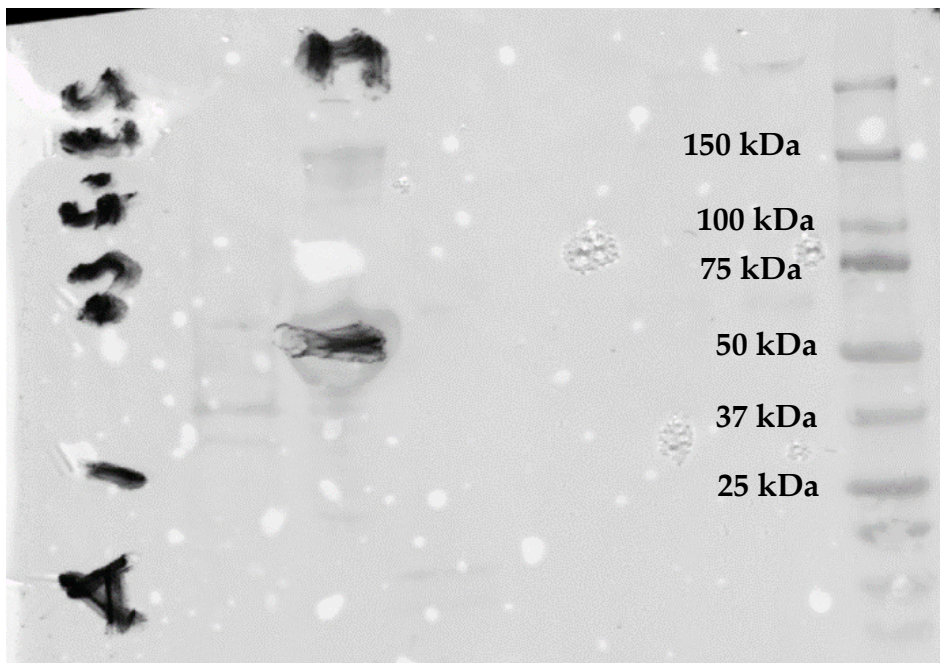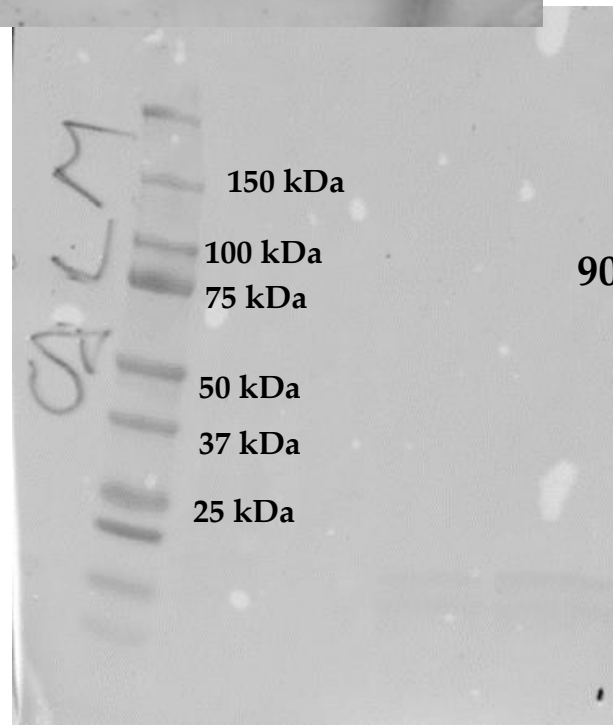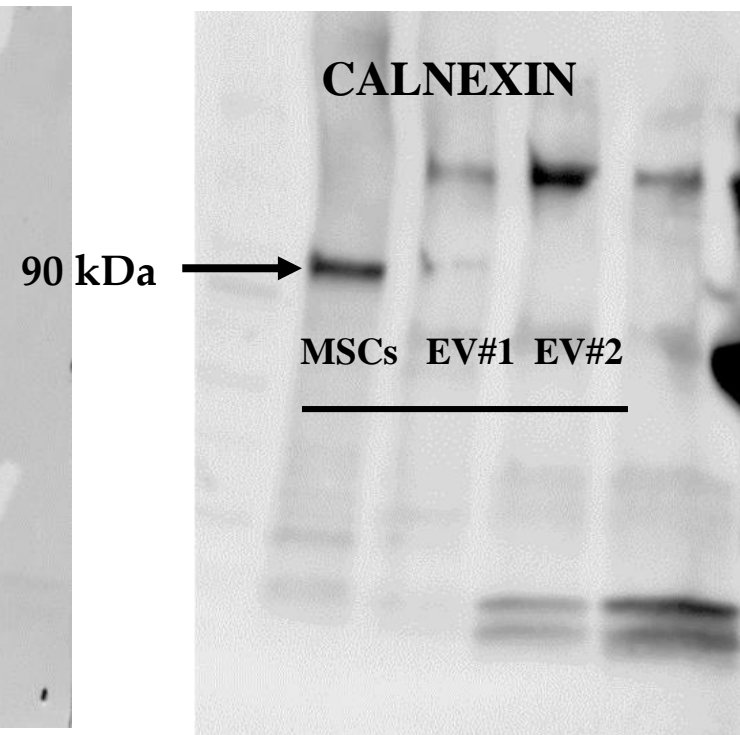

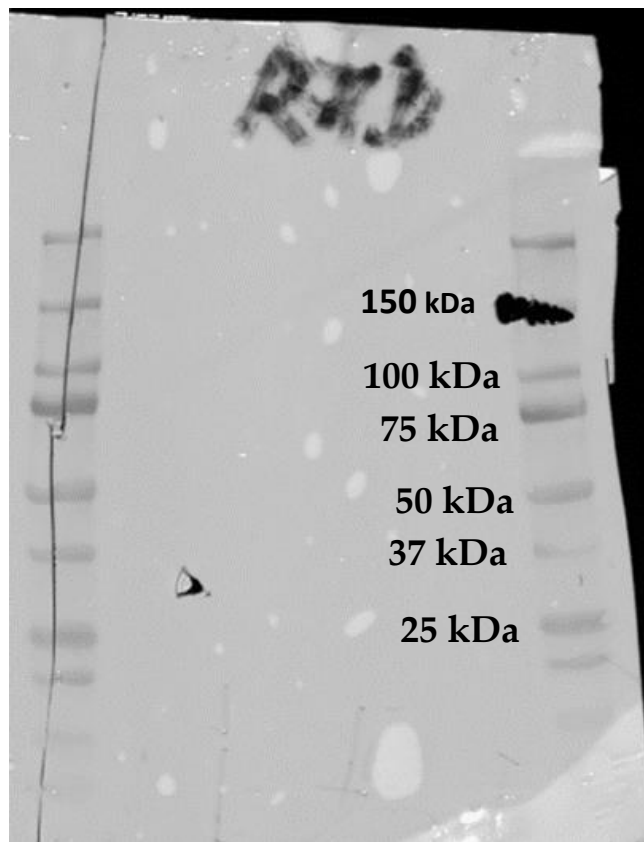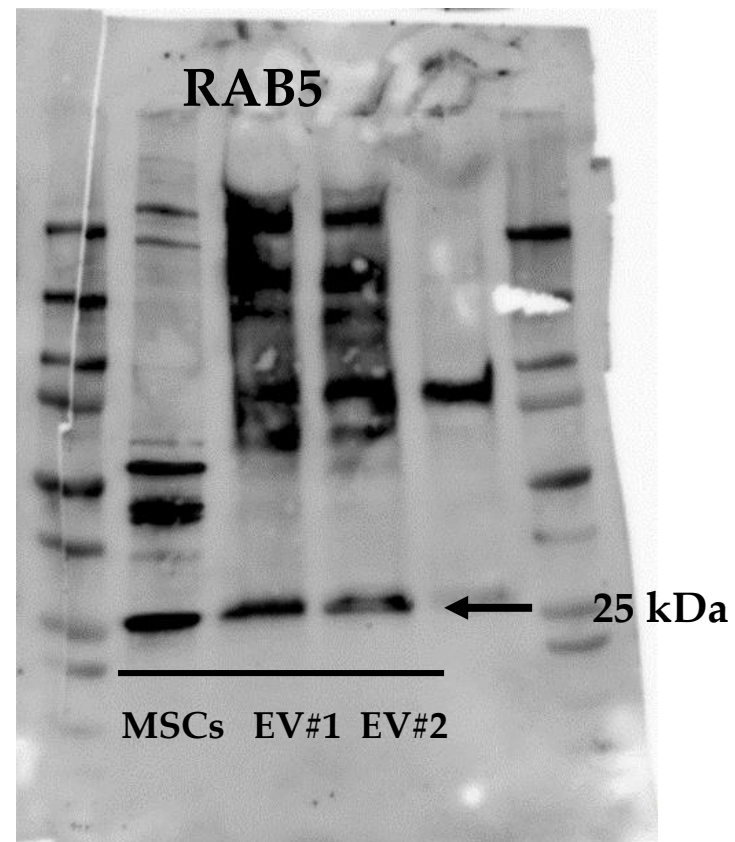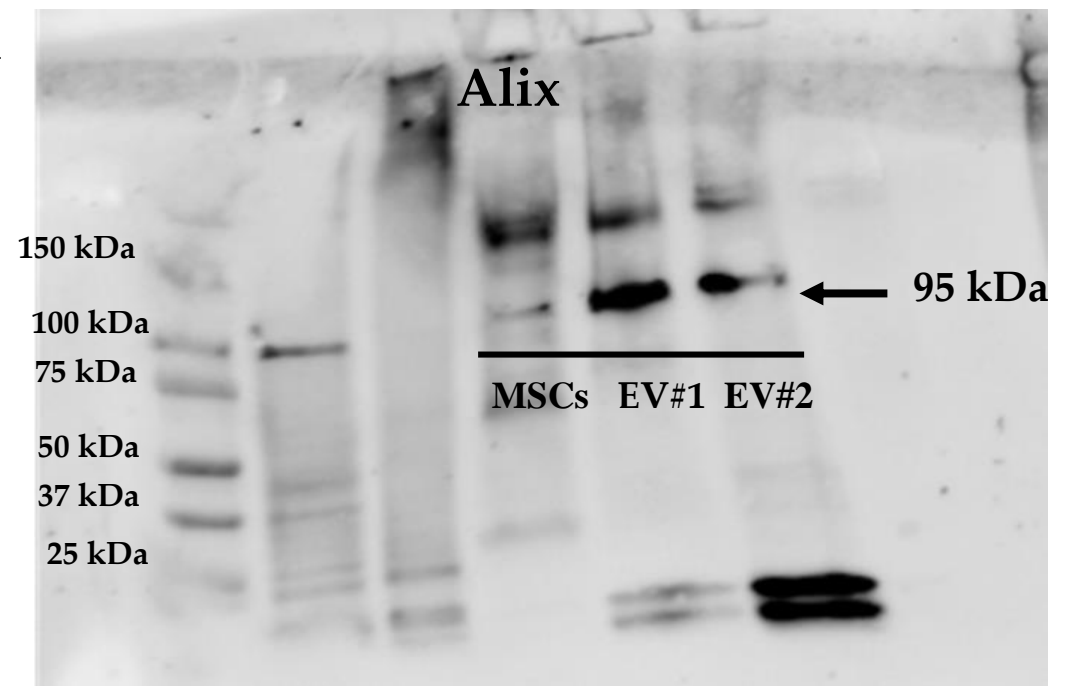

Supplement: Supplementary file 1 [file ijms-22-02045-s001.zip › Figure S1.pdf]

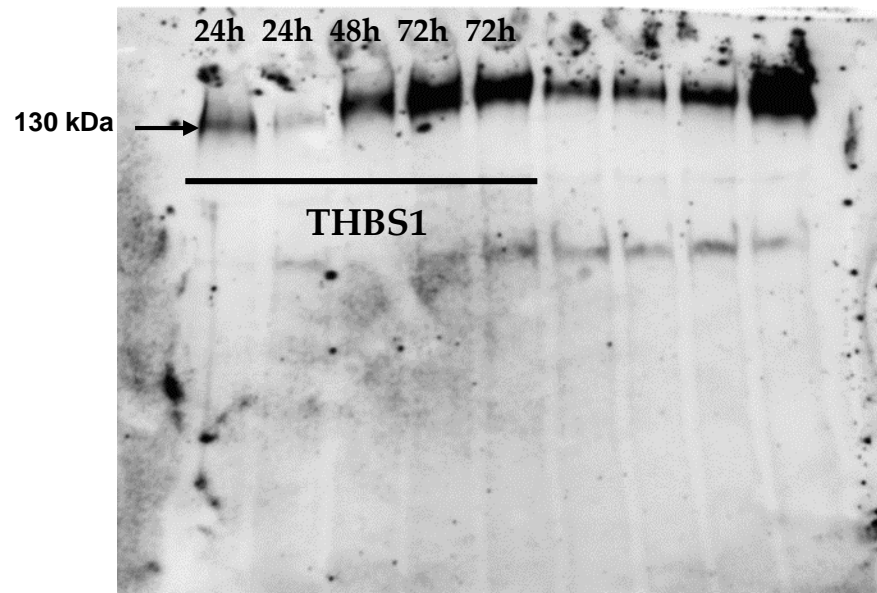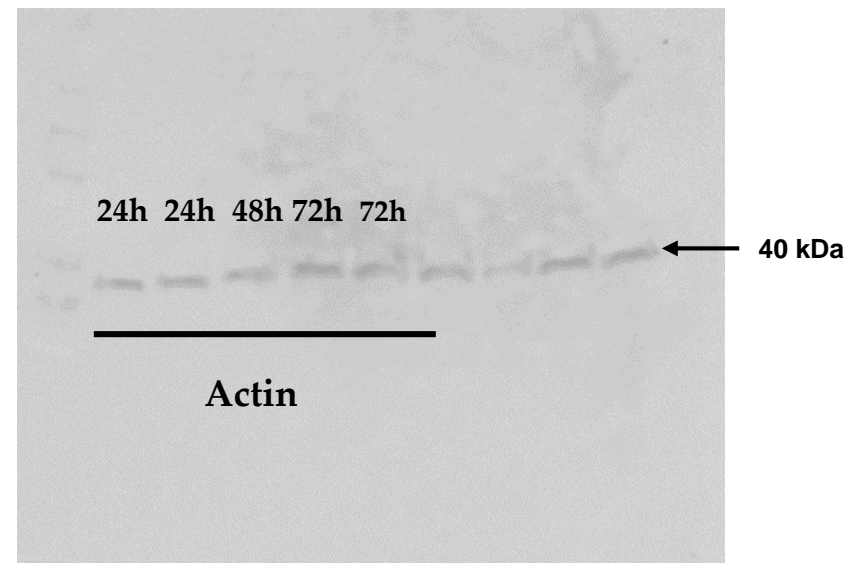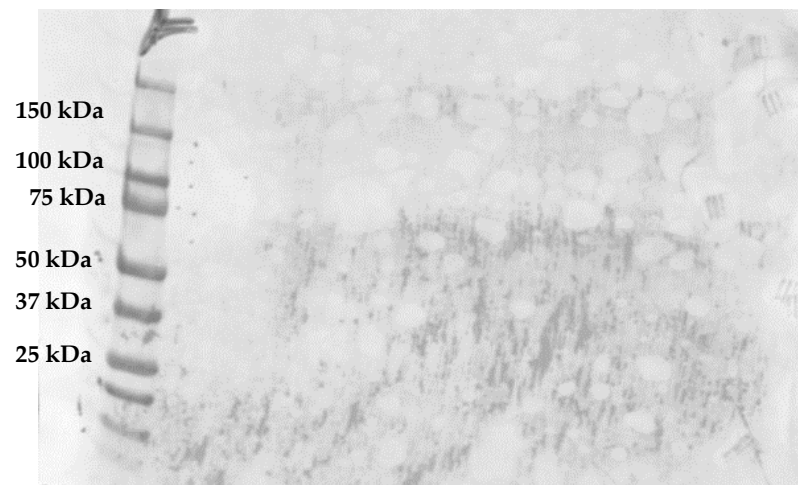

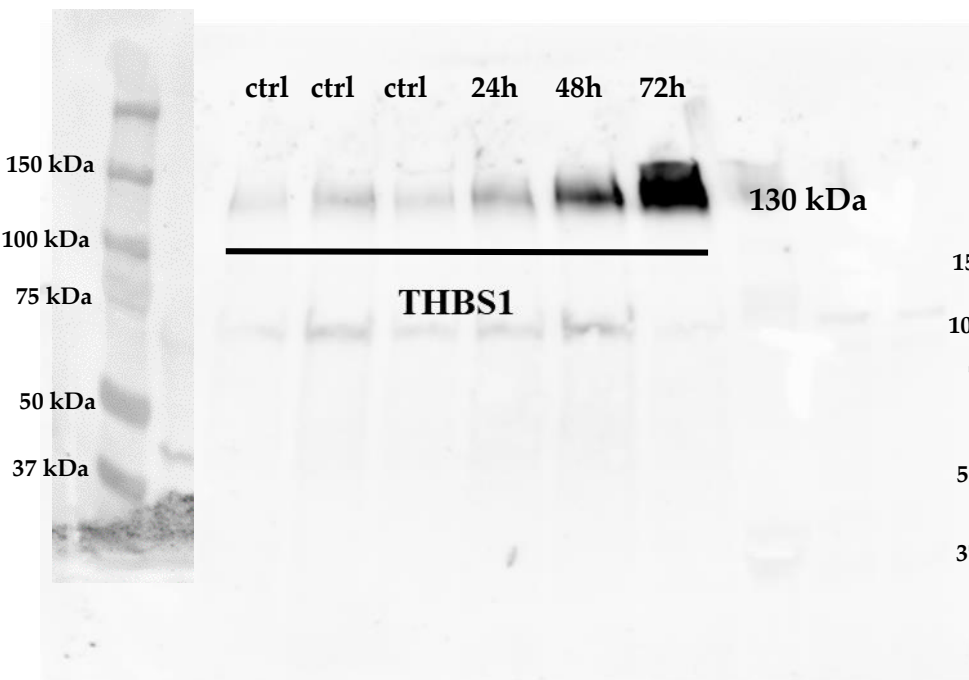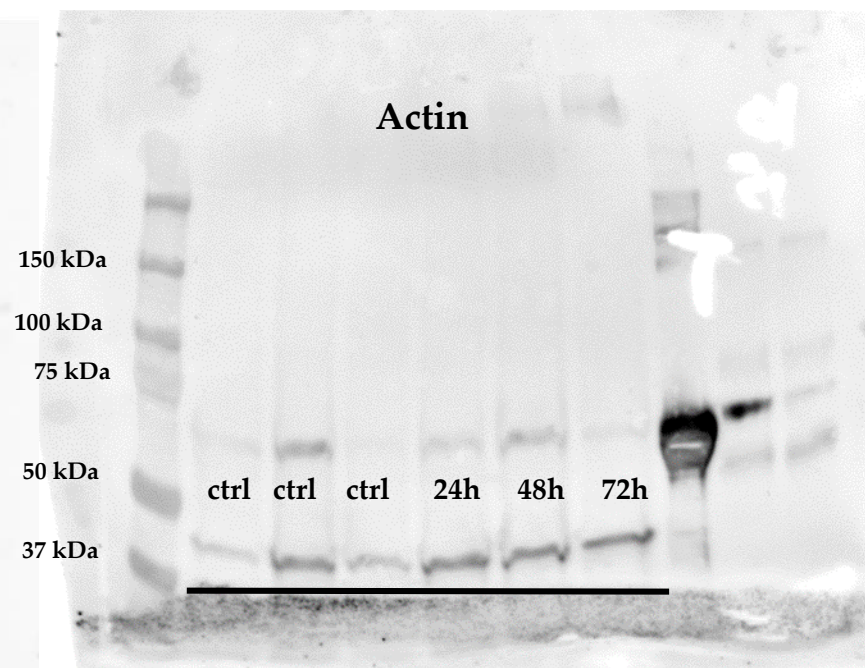

Supplement: Supplementary file 1 [file ijms-22-02045-s001.zip › Figure S2.pdf]
